# Neurofibromatosis Type 1: Clinical and Molecular Characterization in a Romanian Cohort of 80 Patients

**DOI:** 10.3390/diagnostics16213618

**Published:** 2026-11-02

**Authors:** Ionut Gavrila, Anca-Lelia Riza, Răzvan Mihail Pleșea, Rebecca-Cristiana Serban, Mihai Gabriel Cucu, Eugenia Roza, Magdalena Budisteanu, Raluca-Ioana Teleanu, Renata Maria Varut, Cristian Gheonea, Florin Burada, Ioana Streata

**Affiliations:** 1 Laboratory of Human Genomics, University of Medicine and Pharmacy of Craiova, 200638 Craiova, Romania; ionut.gavrila@umfcv.ro (I.G.); anca.riza@umfcv.ro (A.-L.R.); razvan.plesea@umfcv.ro (R.M.P.); rebecca.serban@umfcv.ro (R.-C.S.); mihai.cucu@umfcv.ro (M.G.C.); ioana.streata@umfcv.ro (I.S.); 2 Human Genomics Laboratory—Research Center for Gastroenterology and Hepatology (CCGH), Craiova— University of Medicine and Pharmacy of Craiova, 200638 Craiova, Romania; 3 Clinical Neuroscience Department, Pediatric Neurology, Faculty of Medicine, “Carol Davila” University of Medicine and Pharmacy, 020021 Bucharest, Romania; roza_eugenia@yahoo.com; 4 Department of Pediatric Neurology, Prof. Dr. Alex. Obregia Clinical Hospital of Psychiatry, 041914 Bucharest, Romania; laborator.cercetare.psihiatrie@spital-obregia.ro; 5 “Dr. Victor Gomoiu” Clinical Children’s Hospital, “Carol Davila” University of Medicine and Pharmacy, 050474 Bucharest, Romania; raluca.teleanu@umfcd.ro; 6 Research Methodology Department, Faculty of Pharmacy, University of Medicine and Pharmacy of Craiova, 200349 Craiova, Romania; renata.varut@umfcv.ro; 7 Department of Pediatrics, University of Medicine and Pharmacy of Craiova, 200349 Dolj, Romania; cristian.gheonea@umfcv.ro

**Keywords:** neurofibromatosis type 1, NF1, next-generation sequencing, MLPA, copy-number variant, genotype–phenotype correlation, Romania

## Abstract

**Background/Objectives:** Neurofibromatosis type 1 (NF1) is an autosomal dominant tumor-predisposition disorder with age-dependent manifestations and marked clinical variability. This study characterized clinical observations and documented molecular findings in a Romanian regional referral cohort. **Methods:** We retrospectively analyzed 80 patients referred between 2017 and 2025 because of a family history of NF1 or suggestive clinical signs. Available next-generation sequencing (NGS) and multiplex ligation-dependent probe amplification (MLPA) results were reviewed, and variants were classified according to ACMG/AMP criteria. **Results:** Pathogenic or likely pathogenic NF1 variants were reported in 15/80 patients (18.75%): 13/66 children (19.7%) and 2/14 adults (14.3%). Five sequence variants and ten copy-number deletions were identified. Clinical observations included pigmentary, neurodevelopmental, skeletal, dysmorphic, and tumor-related findings. Testing availability and clinical documentation were heterogeneous. **Conclusions:** The detected molecular spectrum included both sequence and dosage variants. Uneven assay availability limits estimation of the incremental yield of combined testing. Complete diagnostic documentation and age-appropriate follow-up are important for interpreting molecular findings and supporting family counseling.

## 1. Introduction

Neurofibromatosis type 1 (NF1; OMIM 162200) is an autosomal dominant multisystem disorder and tumor-predisposition syndrome caused by heterozygous pathogenic variants in NF1. The gene encodes neurofibromin, a GTPase-activating protein that negatively regulates RAS signaling. Reduced neurofibromin activity promotes persistent RAS–MAPK pathway activation and contributes to pigmentary, neurologic, skeletal, vascular, and neoplastic manifestations [1,2]. Broader translational reviews further emphasize the tumor-suppressor functions of neurofibromin across cellular contexts, while experimental work links its Ras-GAP activity to neuronal differentiation and neurite development [3,4].

The relationship between NF1 variation and clinical expression is complicated by the different biological processes contributing to individual manifestations. Pigmentary signs, developmental difficulties, skeletal abnormalities, and tumors cannot be treated as interchangeable measures of severity. Their clinical importance also changes with age. A child with conspicuous café-au-lait macules may have substantial developmental needs even in the absence of a documented tumor, whereas an adult may require assessment of pain, neurologic change, or a growing lesion. Clinical characterization therefore benefits from describing the affected domains separately rather than reducing the phenotype to a single impression of mild or severe disease [1,3].

Clinical expression is age-dependent and highly variable. Café-au-lait macules and skinfold freckling often appear early, whereas cutaneous neurofibromas accumulate mainly later in life. Optic pathway glioma, plexiform neurofibroma, learning difficulties, skeletal dysplasia, vasculopathy, and malignant peripheral nerve sheath tumor contribute substantially to morbidity. Revised international criteria incorporate both clinical findings and a heterozygous pathogenic NF1 variant, while emphasizing the differential diagnosis of Legius syndrome in young patients with pigmentary findings alone [5].

A further diagnostic challenge is the interpretation of incomplete presentations. Pigmentary findings may precede other diagnostic features, and a family history recorded as café-au-lait macules does not necessarily establish that the relative fulfills NF1 criteria. The revised consensus criteria provide a framework for integrating phenotype, family history, and molecular evidence, but their application still requires appropriately documented examinations [5]. In referral practice, the distinction between a suggestive finding and a confirmed diagnostic criterion is particularly relevant when comparing children of different ages or assessing several members of the same family.

Because NF1 is a tumor-suppressor gene with a broad mutational spectrum, molecular diagnosis may require complementary methods. Sequencing detects single-nucleotide variants and small insertions/deletions, whereas dosage analysis detects exon-level or whole-gene copy-number variants that may be missed by routine sequence analysis [6,7]. Molecular confirmation is particularly useful in young children with incomplete criteria, in atypical presentations, and for cascade or reproductive testing. Large diagnostic series also show that the NF1 mutational spectrum is widely dispersed and that combined DNA, dosage, and RNA-based approaches can improve detection of splice-altering and other difficult-to-detect variants [8].

The interpretation of a laboratory result also depends on the question addressed by the assay. A negative dosage study does not exclude a sequence variant, and a negative sequencing result does not establish that every possible structural or mosaic alteration has been excluded. Clinical suspicion and the scope of completed testing must therefore be considered together. This distinction is essential in retrospective series, where an empty result field may reflect unavailable documentation rather than a true negative result. Describing both the molecular spectrum and the completeness of testing helps define what can reasonably be concluded from a regional referral population [7,8].

This study characterized the clinical observations, documented molecular investigations, and pathogenic or likely pathogenic NF1 findings in 80 patients referred to a regional medical genetics center in Romania. The analysis focused on the spectrum of detected variants and the completeness of testing in routine referral practice.

## 2. Materials and Methods

### 2.1. Study Design and Participants

This retrospective referral-cohort study included 80 patients evaluated at the Regional Center for Medical Genetics Craiova, Dolj, Romania, between 2017 and 2025. Referral indications comprised a family history of NF1 or clinical findings suggestive of the disorder. The cohort included 66 children aged 0–17 years and 14 adults aged 18 years or older. Clinical assessment was guided by established diagnostic criteria, including the revised international consensus criteria [5].

Recorded variables included age, recorded sex, personal and family history, clinical phenotype, diagnostic procedures, lesion type, and available molecular findings. Pre-test and post-test genetic counseling was offered according to standards of care. The study was descriptive; referral criteria were broader than a confirmed clinical diagnosis of NF1.

Clinical observations were extracted from the referral indication and the dedicated clinical-manifestations field. A finding was counted once per patient within each domain when explicitly documented in either source; patients could contribute to multiple domains. Unmentioned findings and blank fields were classified as undocumented, rather than absent. Family history descriptions were distinguished from findings in the referred patient, and suspected lesions were retained separately from confirmed diagnoses.

Recorded ages were used for demographic grouping. Dates of individual clinical observations were not consistently available, preventing alignment of every manifestation with age at examination. Clinical-criteria flags were retained as recorded and were not equivalent to an independently verified criterion-by-criterion diagnosis. Tumor histology, symptom onset, and progression were not inferred from brief referral descriptions.

### 2.2. Next-Generation Sequencing

Genomic DNA was extracted from peripheral blood samples. Whole-exome sequencing was performed using the AmpliSeq for Illumina Exome Panel, with library preparation carried out using the AmpliSeq Library PLUS for Illumina Kit, according to the manufacturer’s instructions. Paired-end 2 × 150 bp reads were generated on a NextSeq 550 IVD platform, with a median target coverage of at least 100×. Reads were mapped to the GRCh37 reference genome using the iGenomes resource bundle and processed through the nf-core/sarek pipeline (version 2.7.1). Sequence analysis was used to identify single-nucleotide variants and small insertions or deletions.

The reported median target coverage summarizes the sequencing dataset; NF1 exon-specific coverage and a validated lower allele-fraction threshold for mosaic variant detection were not available for this retrospective analysis. Deep intronic and regulatory regions and low-level mosaicism were not systematically assessed within the reported exome workflow.

### 2.3. Copy-Number Analysis

NF1 exon-level deletions and duplications were assessed using SALSA MLPA probemixes P081 NF1 MIX1 and P082 NF1 MIX2 (MRC-Holland, Amsterdam, The Netherlands), following the manufacturer’s protocol. Fragment analysis was performed on an ABI PRISM 3730 Genetic Analyzer (Applied Biosystems), and dosage data were evaluated using Coffalyser.Net. The complementary use of sequence and dosage analysis follows established molecular diagnostic strategies for NF1 [7].

Assay availability was assessed from documented results. Molecular investigations were not performed uniformly across the cohort. Individual diagnostic considerations and laboratory availability may have influenced assay selection, but the reasons for using MLPA alone or NGS alone were not consistently recorded and could not be established retrospectively. An unavailable result could reflect either an unperformed test or missing documentation.

### 2.4. Variant Annotation and Classification

Variants were annotated with the Ensembl Variant Effect Predictor and supporting plugins [9]. Pathogenicity was assigned using the five-tier ACMG/AMP framework [10]. Interpretation incorporated population frequency, predicted molecular consequence, published evidence, clinical databases including ClinVar [11], and segregation data when available. Only pathogenic and likely pathogenic findings are reported in the principal analyses. Broader conceptual extensions of the ACMG categories underscore the importance of interpreting evidence in its gene- and disease-specific context [12].

Variant interpretation also requires a clear distinction between a molecular consequence and its demonstrated clinical significance. A predicted splice effect does not, by itself, establish the resulting transcript, and a missense substitution cannot be assigned a specific clinical course solely from its protein position. Variants of uncertain significance should remain separate from diagnostic findings. When clinical databases are consulted, the relevant transcript, disease assertion, evidence, and review status must be considered alongside the laboratory classification and the patient’s phenotype [10,11].

### 2.5. Statistical Analysis

Categorical variables were summarized as counts and percentages with explicit denominators. Molecular detection among all referrals was distinguished from the proportion with a pathogenic or likely pathogenic finding among patients with a documented result from the relevant assay. Missing results were retained as a separate category. Age and sex summaries were descriptive; no inferential comparisons or formal genotype–phenotype association tests were performed because the subgroups were small and assessments were heterogeneous.

Clinical observations could not be reliably cross-tabulated by age at examination or sex because these links were unavailable in the aggregate summaries. Standardized follow-up was not consistently documented, so changes in phenotype and the emergence of additional complications were not assessed longitudinally.

Clinical counts represent documented observations among referrals, rather than the prevalence of systematically examined manifestations. No missing observations were imputed. Patients with findings in more than one domain were counted in each applicable domain, and the inclusion of relatives limits assumptions of statistical independence.

## 3. Results

### 3.1. Cohort Characteristics and Molecular Diagnostic Yield

The cohort comprised 80 patients, including 46 males and 34 females. Pathogenic or likely pathogenic NF1 variants were detected in 15 patients (18.75%). Thirteen of 66 children were molecularly positive (19.7%), compared with 2 of 14 adults (14.3%) (Table 1). Because the cohort included patients referred on the basis of either family history or suggestive signs, these proportions represent the yield in this referral population and should not be interpreted as analytical sensitivity.

**Figure 1:**
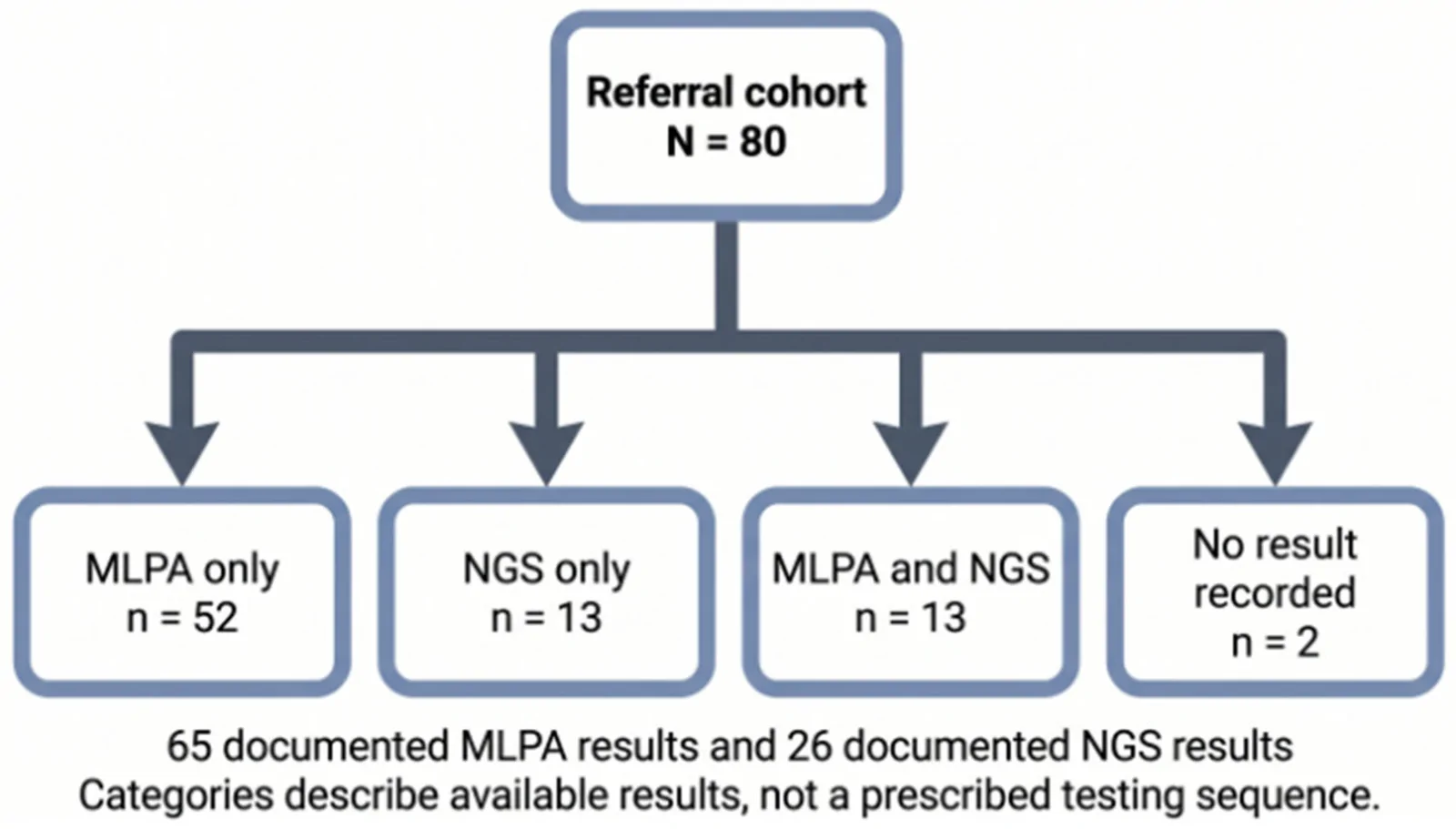
Documented molecular-test availability in the 80-patient referral cohort. The four categories describe available results; an unrecorded result does not establish whether testing was performed. Created in BioRender.com Varut, R. (2026), https://app.biorender.com/illustrations/670666effff8cafd86571cff (accessed on 28 October 2026).

MLPA results were documented for 65 patients and NGS results for 26, including 13 with both modalities. Thus, 52 patients had MLPA alone documented, 13 had NGS alone, 13 had both results, and 2 had neither result documented (Table 2; Figure 1). A pathogenic or likely pathogenic finding was reported in 10/65 patients with MLPA results (15.4%) and 5/26 with NGS results (19.2%). Among the 13 patients with results from both assays, four had a pathogenic or likely pathogenic NF1 finding. Based on assay-specific totals, two findings were identified by MLPA and two by NGS. These data describe the distribution of documented molecular findings and do not establish the incremental diagnostic yield of a standardized sequential testing strategy. These assay-specific proportions have different, overlapping denominators. Positive and negative molecular findings were summarized according to documented testing combinations (Table 2). However, the absence of a reported pathogenic or likely pathogenic variant does not exclude NF1, particularly in patients with incomplete molecular testing or persistent clinical suspicion.

A family history suggestive of NF1 was documented in eight patients; four had genetically confirmed affected relatives. Family history supported referral and cascade evaluation but did not replace molecular confirmation.

### 3.2. Sequence Variants Identified by NGS

Five distinct pathogenic or likely pathogenic sequence variants were detected, each in one patient: 5/26 patients with documented NGS results (19.2%) and 5/80 of all referrals (6.25%) (Table 3). These comprised two frameshift deletions, two missense substitutions, and one recurrent in-frame deletion.

### 3.3. Copy-Number Variants Identified by MLPA

MLPA identified ten pathogenic copy-number alterations (Table 4). Six involved a 17q11.2/whole-gene NF1 deletion, while four were intragenic exon deletions. Thus, dosage analysis accounted for two-thirds of the molecularly positive findings in this selected cohort.

### 3.4. Clinical Observations

Café-au-lait macules were the most consistently documented feature among molecularly positive patients. Other findings included axillary or inguinal freckling, neurofibromas, neurodevelopmental impairment, craniofacial dysmorphism, skeletal abnormalities, hypotonia, and growth disturbances. The six patients with 17q11.2/whole-gene deletions showed heterogeneous involvement; documented features included pigmentary findings, neurofibromas, developmental or intellectual impairment, dysmorphism, and, in one patient, renal and urogenital malformations.

Café-au-lait macules were documented in at least 77 records and axillary or inguinal freckling in 18 records. Selected observations included speech or language difficulties and scoliosis or kyphosis in 6 records each; macrocephaly and pectus abnormalities in 4 each; optic nerve glioma and epileptic manifestations in 3 each; and Lisch nodules, attention-deficit/hyperactivity disorder (ADHD), autism spectrum disorder, hypotonia, ligamentous hyperlaxity, and hearing impairment in 2 each. Table 5 summarizes clinical domains using referral and clinical narratives. These documentation counts do not represent systematically assessed prevalence.

The two truncating sequence variants were associated with relatively mild documented phenotypes despite their predicted loss-of-function effect. The p.Cys1016Arg and p.Lys1457Gln missense variants occurred in patients with multisystem or neurodevelopmental findings. The patient carrying p.Met992del presented mainly with café-au-lait macules and axillary freckling. These observations are descriptive because each sequence variant was identified in only one patient and phenotyping was retrospective.

The referral narratives described a wider range of neurologic and ophthalmologic findings across the cohort. These included visual impairment, strabismus, refractive errors, congenital ocular abnormalities, optic nerve glioma, seizures, and intracranial lesions involving structures such as the thalamus, globus pallidus, cerebral peduncle, or hypothalamic region. Some records included a named diagnosis, whereas others contained only an imaging or clinical description. The latter were not considered sufficient to establish a specific tumor type. These observations illustrate the need to distinguish functional impairment, structural abnormalities, and confirmed neoplasia when summarizing NF1-related morbidity.

Developmental descriptions included speech and language difficulties, motor delay, global developmental disorder, intellectual disability, attention-deficit/hyperactivity disorder, and autism spectrum disorder. These findings appeared in different combinations and were not confined to a single molecular category. Formal neuropsychological scores, standardized developmental instruments, and consistent assessment ages were not recorded. Consequently, the narratives describe the types of difficulties encountered in the referral population without establishing their comparative prevalence or a relationship between a particular variant and cognitive outcome.

Skeletal and growth-related observations included scoliosis or kyphoscoliosis, pectus abnormalities, ligamentous laxity, hypotonia, delayed skeletal maturation, and impaired linear or weight growth. More detailed individual records also described vertebral scalloping, spina bifida occulta, or foot abnormalities. These findings were retained as clinical observations and were not automatically classified as the specific osseous lesions required by diagnostic criteria. In the child with p.Met992del, growth and nutritional concerns, vitamin D deficiency, and mild aortic regurgitation accompanied the pigmentary presentation. Their coexistence does not establish that each feature was caused by this variant.

Adult referral records included clinically important findings extending beyond cutaneous manifestations. One described a history of operated stage IIIA breast cancer, and another a duodenal mass without a documented histologic subtype. A separate presentation combined a cerebellopontine-angle schwannoma with marked hearing impairment, balance disturbance, and facial paresis. Such descriptions require individual interpretation and, where appropriate, consideration of an alternative or additional diagnosis. They do not provide a basis for estimating cancer incidence or attributing every recorded tumor to NF1.

Clinical concerns also persisted in individuals without a documented positive molecular result. Records in this group included optic nerve glioma, a thoracic plexiform neurofibroma, cutaneous neurofibromas, seizures, and spinal or developmental abnormalities. A negative individual assay therefore did not resolve every clinical presentation. The relevance of these cases lies in the coexistence of continued clinical suspicion and incomplete or negative molecular evidence, rather than in evidence that the reported manifestations were absent or unrelated. Table 5 summarizes the principal domains represented in the clinical narratives.

Clinical manifestations were explicitly recorded in the dedicated clinical field for 41 patients. However, additional clinically relevant information was sometimes available in the referral indication, including pigmentary findings, skinfold freckling, tumor descriptions, or affected relatives. Therefore, a blank clinical-manifestations field was not interpreted as absence of clinical involvement. For the descriptive analysis, information from both fields was considered, while referral findings were kept distinct from confirmed diagnoses.

## 4. Discussion

This regional cohort describes NF1 molecular findings within a heterogeneous referral pathway. Detection proportions depend on referral criteria, the assays completed, and the availability of their results. Studies restricted to clinically established NF1 and using comprehensive DNA, dosage, or RNA testing provide useful diagnostic benchmarks, but their yields are not directly comparable with a partially investigated referral population [7,8,13]. A Japanese hereditary-tumor cohort, for example, identified pathogenic NF1 variants in 36 of 44 patients after sequencing and copy-number analysis, illustrating the importance of defining both the population and the testing pathway [14].

The unequal availability of sequencing and dosage results may contribute to the predominance of copy-number findings among positive cases. The coexistence of sequence and dosage variants supports the relevance of both variant classes in diagnostic practice; however, the incremental yield of a uniform combined strategy requires paired testing with a defined order and complete outcome ascertainment. A negative result from one assay leaves variant classes outside its validated scope unresolved.

Exome-based sequencing can miss deep intronic and regulatory variants, complex rearrangements, or low-level mosaicism, depending on coverage, bioinformatic filters, and the tissue examined. Transcript analysis can reveal splice abnormalities and allele-specific effects that are not apparent from routine DNA testing [8,13,15]. In 2026, Doolan et al. reported five children with clinically established NF1 and negative DNA-based NGS whose diagnoses were resolved by cDNA testing [16]. A 2025 long-read study demonstrated additional characterization of intronic and structural variation and reported a 92.1% yield among 126 pediatric probands with NF1 [17]. These studies support phenotype-directed investigation of unresolved cases, while the causes of negative results in the present cohort remain unverified.

Pigmentary presentations also require differential diagnosis. Café-au-lait macules, skinfold freckling, and a family history of similar findings may occur in SPRED1-related Legius syndrome, particularly before other age-dependent NF1 manifestations emerge [5]. Legius syndrome was considered clinically, but systematic SPRED1 testing was not documented in this cohort. This differential diagnosis therefore remains unresolved in patients with predominantly pigmentary features and no molecular NF1 confirmation. Reassessment of the phenotype and the scope of prior testing can guide further gene-specific or panel-based investigation.

Most individual NF1 variants have limited prognostic precision, despite established associations for selected recurrent variants [18,19]. The predominantly pigmentary phenotype associated with p.Met992del is supported by larger series and longitudinal observations [20,21]. Recent domain-based analyses also demonstrate the scale and phenotypic detail required to investigate such relationships [22]. Single-patient observations in this cohort cannot establish variant-specific severity or explain every co-occurring feature; clinical surveillance therefore remains guided by age and manifestations.

Large NF1 deletions may encompass neighboring genes and are associated with a broader neurodevelopmental, dysmorphic, and tumor-related phenotype [23,24]. Here, the deleted genomic interval refers to the segment of chromosome 17q11.2 removed by a deletion, including its breakpoints and any neighboring genes involved. MLPA establishes dosage loss at the interrogated probes but does not define that full interval. Further breakpoint-resolving analysis may therefore be appropriate when the extent of a suspected contiguous-gene deletion would inform counseling or surveillance.

Age-dependent expression is central to interpreting this predominantly pediatric cohort. Skinfold freckling may emerge during childhood, whereas cutaneous neurofibromas and several tumor complications become more evident later. Brief retrospective records can also undercapture signs that were not systematically examined. Consequently, a low documented freckling count cannot be attributed to age alone, and childhood observations do not describe lifetime disease burden. Recent large-scale phenotyping studies further show that population composition and ascertainment influence observed clinical distributions [25]. Pediatric follow-up should integrate ophthalmologic, developmental, skeletal, skin, and blood-pressure assessment with routine preventive care [26,27,28,29,30]. Adult assessment additionally addresses changing lesions, pain, neurologic symptoms, vascular complications, and malignancy risks [31,32,33].

Molecular confirmation supports targeted testing of relatives and reproductive counseling. Family reports of pigmentary findings warrant evaluation but do not establish a segregating pathogenic variant. Complete pedigrees and parental results are needed to distinguish inherited from de novo disease. The retrospective records also provide insufficient longitudinal information to determine the age at onset or progression of complications.

Advances in treatment increase the practical importance of accurate diagnosis and coordinated follow-up. Selumetinib has demonstrated activity in children with symptomatic, inoperable NF1-associated plexiform neurofibromas [34]. Eligibility depends on the lesion, symptoms, progression, and multidisciplinary assessment rather than on the germline variant alone; treatment effectiveness was not evaluated in this cohort.

Regional data can describe how diagnostic pathways operate within a specific service. Together with the recent Romanian cohort from Iași [35], the present observations support harmonized recording of referral indications, assessment dates, individual diagnostic criteria, assay completion, and molecular nomenclature. Their contribution is local characterization and identification of documentation gaps; estimates of national variant frequencies or comparative service performance would require coordinated sampling and standardized testing.

The retrospective design, heterogeneous referral criteria, incomplete testing records, and nonuniform phenotyping limit generalizability. Exact assessment ages, feature-specific examination denominators, and cross-tabulations linking clinical and molecular findings to age and sex were unavailable. Standardized follow-up was not consistently documented, preventing reliable assessment of progression or newly developing complications. The small adult and variant-defined subgroups further constrain comparative interpretation. Systematic SPRED1 analysis, transcript studies, validated low-level mosaicism assessment, and breakpoint-resolving data were unavailable within the reported workflow. Prospective evaluation with complete assay records and standardized longitudinal phenotyping would strengthen diagnostic and prognostic interpretation.

## 5. Conclusions

This Romanian referral cohort documents both NF1 sequence variants and copy-number deletions in patients with heterogeneous clinical presentations. Incomplete and uneven assay availability limits interpretation of the overall detection proportion and of the incremental benefit of combined testing. Standardized phenotyping, complete molecular investigation where indicated, and longitudinal follow-up would strengthen diagnostic clarification and family counseling.

## Figures and Tables

**Table 1:** Demographic distribution and molecular findings in the referral cohort.

Age Group	Patients, *n*	Male, *n*	Female, *n*	P/LP NF1 Variant, *n* (%)
0–17 years	66	40	26	13 (19.7)
≥18 years	14	6	8	2 (14.3)
Total	80	46	34	15 (18.75)

P/LP, pathogenic or likely pathogenic. Percentages use the total in each age group. Sex-specific counts of molecularly positive patients were unavailable in the aggregate summaries; no sex-effect comparison was performed.

**Table 2:** Documented test availability and assay-specific molecular findings. The first four rows represent mutually exclusive testing groups and sum to 80 patients. The final two rows provide assay-specific summaries with overlapping denominators and should not be added together. The absence of a reported pathogenic or likely pathogenic variant does not exclude NF1 or establish that all relevant variant classes were investigated.

Result Availability or Assay	Patients, *n*	P/LP Finding, *n*	No P/LP Finding Reported, *n*
MLPA alone	52	8	44
NGS alone	13	3	10
Both results	13	4	9
Neither result	2	Not assessable	Not assessable
All MLPA results	65	10	55
All NGS results	26	5	21

**Table 3:** Pathogenic and likely pathogenic NF1 sequence variants detected by NGS.

Reference Sequence and Variant	Molecular Consequence	Classification	Cases, *n*	Documented Clinical Features
NM_001042492.3:c.2082del; p.(Leu695CysfsTer53)	Frameshift	Pathogenic	1	Café-au-lait macules; cutaneous neurofibromas
NM_001042492.3:c.1466_1473del; p.(Tyr489SerfsTer19)	Frameshift	Likely pathogenic	1	Café-au-lait macules; cutaneous neurofibromas
NM_001042492.3:c.3046T>C; p.(Cys1016Arg)	Missense	Pathogenic	1	Macrocephaly; ocular, craniofacial, skeletal, and growth findings
NM_001042492.3:c.4369A>C; p.(Lys1457Gln)	Missense	Pathogenic	1	Pigmentary, neurodevelopmental, microcephalic, and skeletal findings
NM_001042492.3:c.2970_2972del; p.(Met992del)	In-frame deletion	Pathogenic	1	Café-au-lait macules and axillary freckling

NGS, next-generation sequencing. Each variant was reported in one patient. Sex-linked variant and phenotype data were unavailable in the aggregate summaries.

**Table 4:** Pathogenic NF1 copy-number variants detected by MLPA.

Copy-Number Finding	Cases, *n*	Classification	Clinical Interpretation
17q11.2/whole-gene NF1 deletion	6	Pathogenic	NF1 microdeletion; may include contiguous genes depending on deletion extent
NF1 exon 23 deletion	1	Pathogenic	Intragenic exon-level deletion
NF1 exon 13 deletion	2	Pathogenic	Intragenic exon-level deletion
NF1 exon 9 deletion	1	Pathogenic	Intragenic exon-level deletion
Total	10	—	—

MLPA, multiplex ligation-dependent probe amplification. Precise genomic breakpoints and sex-specific variant counts were unavailable.

**Table 5:** Clinical domains represented in the referral and clinical narratives.

Clinical Domain	Selected Findings Documented in the Referral or Clinical Narratives	Records with Documented Findings, *n*
Ophthalmologic	Visual impairment, ocular abnormalities, optic glioma, Lisch nodules	11
Neurologic	Seizures, intracranial lesions, cranial/peripheral nerve involvement	9
Craniofacial and dysmorphic	Facial dysmorphism or asymmetry and craniofacial abnormalities	12
Developmental and behavioral	Speech or developmental difficulties, intellectual disability, ADHD, autism	12
Skeletal and muscular	Spinal or chest-wall abnormalities, laxity, hypotonia, foot findings	11
Growth and nutrition	Growth impairment, nutritional problems, vitamin D deficiency	3
Tumor-related observations	Neurofibromas, optic glioma, and other recorded tumor-related lesions	15

Counts refer to the full referral cohort (N = 80) and combine referral indications with clinical notes. Each patient is counted once per domain and may contribute to several domains. Feature-specific examination denominators and age- or sex-linked domain counts were unavailable; unmentioned findings were not considered absent. Suspected lesions were not treated as histologically confirmed tumors. ADHD denotes attention-deficit/hyperactivity disorder.

## Data Availability

The original contributions presented in this study are included in the article. Further inquiries can be directed to the corresponding author.

## Abbreviations

ACMG	American College of Medical Genetics and Genomics
AMP	Association for Molecular Pathology
CNV	Copy-number variant
MLPA	Multiplex ligation-dependent probe amplification
MPNST	Malignant peripheral nerve sheath tumor
NGS	Next-generation sequencing
NF1	Neurofibromatosis type 1
P/LP	Pathogenic or likely pathogenic
VEP	Variant Effect Predictor
